# DNA methylation dynamics during the mammalian life cycle

**DOI:** 10.1098/rstb.2011.0328

**Published:** 2013-01-05

**Authors:** Jamie A. Hackett, M. Azim Surani

**Affiliations:** Wellcome Trust/Cancer Research UK Gurdon Institute, University of Cambridge, Cambridge CB2 1QN, UK

**Keywords:** epigenetics, DNA methylation, reprogramming, PGC, bisulphite

## Abstract

DNA methylation is dynamically remodelled during the mammalian life cycle through distinct phases of reprogramming and de novo methylation. These events enable the acquisition of cellular potential followed by the maintenance of lineage-restricted cell identity, respectively, a process that defines the life cycle through successive generations. DNA methylation contributes to the epigenetic regulation of many key developmental processes including genomic imprinting, X-inactivation, genome stability and gene regulation. Emerging sequencing technologies have led to recent insights into the dynamic distribution of DNA methylation during development and the role of this epigenetic mark within distinct genomic contexts, such as at promoters, exons or imprinted control regions. Additionally, there is a better understanding of the mechanistic basis of DNA demethylation during epigenetic reprogramming in primordial germ cells and during pre-implantation development. Here, we discuss our current understanding of the developmental roles and dynamics of this key epigenetic system.

## 1. Introduction

The mammalian life cycle is characterized by extended phases of cellular differentiation, followed by distinct periods of reprogramming in germ cells and the zygote, which resets genomic potential to restart the cycle. Cell differentiation is directed by transcription factor (TF) networks, which establish gene expression patterns in response to developmental cues. Once established, core TF networks maintain robust lineage-restriction and ensure unidirectional development towards defined differentiated cell-types. In parallel, epigenetic mechanisms act to reinforce cell-fate decisions and establish barriers against reversion to preceding cellular states [1]. Multiple epigenetic systems including DNA methylation, histone modifications and small RNAs function both cooperatively and autonomously to establish the epigenome that propagates gene expression profiles through mitosis/meiosis. Disruption of these epigenetic mechanisms can lead to loss of cell identity, cellular transformation or disease, and is generally incompatible with normal development. However, at two unique junctures of mammalian development—in primordial germ cells (PGCs) and pre-implantation embryos—the epigenome is comprehensively reprogrammed towards a basal state to enable relaxation of the epigenetic constraints imposed on cell potential and ultimately, resetting of the life cycle [2]. Of particular recent interest are the mechanisms which erase and establish DNA methylation during development and the roles of these epigenetic events.

In mammals, DNA methylation (5mC) is essential for embryonic development and plays important roles in genomic imprinting, transposon silencing, X-inactivation and gene repression [3]. During development 5mC patterns are established by the de novo methyltransferases DNMT3A and DNMT3B, and subsequently preserved through cell divisions by DNMT1 [4,5]. As such, DNA methylation represents a true epigenetic mechanism because 5mC is faithfully maintained in the absence of the inducing signal. DNA methylation primarily occurs at cytosine residues within CpG dinucleotides, although it is becoming increasingly evident that there may be significant methylation in non-CpG (CpH) contexts, at least in pluripotent cell-types and oocytes [6–8]. Owing to the inherent mutagenicity of methylated cytosine by deamination to thymidine, the bulk of the genome is depleted of CpG motifs, with the exception of distinct regions known as CpG islands (CGIs), which are often associated with promoters. In general, the vast majority of CGIs remain unmethylated during development, whereas most other CpG sites throughout the genome are methylated irrespective of genomic context, suggesting that methylation is the default state and that CGIs are exceptions to the rule [9,10]. Notably, TET-family proteins can modify CpG methylation to 5-hydroxymethylcytosine (5hmC), 5-formylcytosine (5fC) and 5-carboxylcytosine (5caC) [11,12], although it is currently unclear whether these methylation derivatives have specific epigenetic roles or act only as a route towards DNA demethylation [13]. Nonetheless, recent studies have begun to unravel many aspects of DNA methylation biology including its dynamic distribution through development, the mechanisms of demethylation, and the role of CpG methylation in regulating gene transcription in distinct genomic contexts.

Here, we focus on the accumulating knowledge of the dynamics and roles of DNA methylation during the mammalian life cycle. A deeper understanding of this key epigenetic system and the mechanisms which establish and erase it may provide opportunities to manipulate cell identity for therapeutic purposes.

## 2. Strategies for profiling DNA methylation

A fundamental requirement to study DNA methylation is the ability to detect and track 5mC genomic levels efficiently and at a suitable resolution. However, unlike the chromatin immunoprecipitation (ChIP) method to detect histone modifications, there is no single preferred method for 5mC analysis, and a number of techniques have been developed that each have strengths and weaknesses. Broadly, genome-wide DNA methylation analysis can be divided into three approaches: (i) chemical (bisulphite, BS) modification, (ii) affinity enrichment or (iii) differential enzymatic digestion. These can be combined and/or coupled with single-locus-, microarray- or deep-sequencing- analysis to generate a vast number of different analytical techniques [14].

The BS methods rely upon the deamination of unmethylated cytosine to uracil after exposure to sodium bisulphite, while methylated cytosines are resistant and can thus be distinguished by direct sequencing [15]. This approach can generate a quantitative measure of modified cytosine across the whole genome (WGBS) at single-base resolution (including non-CpG methylation) and can be performed on relatively low input DNA quantities (more than 0.5 ng) [16,17]. In addition, BS sequencing is sensitive to relatively small changes (5–10%) in methylation and has thus been considered the ‘gold standard’. However, the method has several drawbacks. First, BS conversion is not specific for precise cytosine modifications, as 5mC is indistinguishable from 5hmC, and unmodified cytosine is indistinguishable from 5caC by current methods [18,19]. A recently developed technique called oxidative bisulphite (oxBS) has partly resolved this issue by specifically identifying 5hmC, although it requires a relatively high amount of input DNA and does not discriminate other cytosine derivatives (5fC and 5caC) [20]. Second, there are potentially significant amplification biases when 5hmC is present, or between differentially methylated amplicons, which could lead to inaccurate quantification owing to preferential exclusion of certain epigenetic states [21,22]. Third, despite recent improvement in deep-sequencing technologies, a genome-wide BS analysis of sufficient depth remains costly. This last point has been addressed by reduced representation bisulphite sequencing (RRBS), which preferentially interrogates CGIs and therefore reduces sequencing expense, but at the cost of reduced coverage, or through the use of infinium methylation arrays which infer the locus methylation state from dual CpG dinucleotides [9,23,24]. Overall, despite some drawbacks, BS approaches coupled with deep-sequencing offer the highest resolution and reproducibility currently available.

Affinity-based methods use specific antibodies or protein interactions to enrich methylated DNA fragments, with the number of subsequent sequencing reads directly related to the underlying methylation level at a locus. Affinity approaches can also be used to determine the overall global level of DNA modification through immunofluorescence, dot-blot or ELISA. Recently, genome-wide methylated DNA immunoprecipitation (meDIP-seq) has become popular in part due to the ability to distinguish 5mC- and 5hmC- profiles with specific antibodies (meDIP & hmeDIP) [25–28]. This is particularly important in studies investigating dynamic DNA demethylation or underlying epigenetic states, as 5hmC is a likely demethylation intermediate [13], and may have a specific epigenetic role [29]. Additionally, (h)meDIP-seq can interrogate both CpG and non-CpG methylation patterns and has recently been optimized for use with low input DNA quantities from rare cell populations [30,31]. Other enrichment methods such as the methylated CGI recovery assay (MIRA) or methyl-binding domain-seq (MBD-seq) can be used to selectively purify only CpG-methylated DNA (rather than CpH methylation) [32,33]. Alternatively, genomic regions that are selectively unmethylated can be assayed using CXXC-affinity purification (CAP-seq) [34]. The resolution of affinity-based methods is dependent on input fragment size, with approximately 150 bp generally the optimal trade-off between resolution and immunoprecipitation efficiency [31]. Notably fragment sizes of more than 500 bp, as used in some studies, can lead to false enrichments over promoter regions, where the first internal exon is often heavily methylated, leading to an apparent upstream promoter peak. A significant drawback of affinity approaches is that they preferentially interrogate CpG-dense regions and that enrichment represents a distribution of DNA modification rather than a quantitative measure [35]. This second issue is compounded by the difficulty in comparing genomic regions of differing CpG density, where the underlying substrate is unevenly distributed, although analytical algorithms have been developed to account for this [36]. Overall, while affinity enrichment does not enable the base-resolution of BS sequencing, the capacity to distinguish cytosine derivatives and the markedly reduced cost currently make it an important complementary technique for genome-wide DNA modification analysis.

The final broad grouping of global DNA methylation analysis methods entail differential enzymatic digestion of methylated and unmethylated regions. Several approaches have been used, such as comprehensive high-throughput analysis for relative methylation (CHARM) and *HpaII* tiny fragment enrichment by ligation-mediated PCR (HELP-seq) [37,38]. In general, enzymatic approaches suffer from the resolution limit imposed by the necessity of specific restriction sites but have found specific niches. In particular, the recent development of the glucosyltransferase assay has permitted quantitative analysis of 5hmC levels at specific loci by differential digestion between *MspI* and *HpaII* [26].

## 3. DNA methylation during the mammalian life cycle

**Figure 1.**
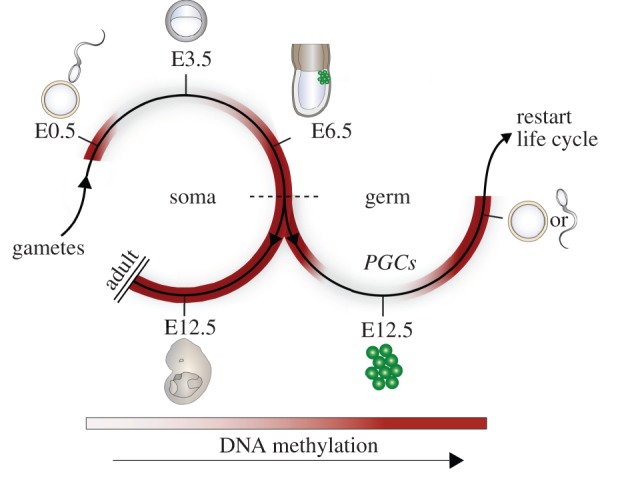
Global DNA methylation dynamics during the life cycle. Upon fertilization genome-wide DNA demethylation occurs in the zygote by conversion to 5hmC on the paternally derived genome and direct passive 5mC depletion of the maternally derived genome. The low-point of global methylation occurs at approximately E3.5 in the ICM, after which global re-methylation begins and reaches near completion by E6.5. At approximately E6.5, cells either continue to develop towards a somatic fate (left) or are specified as primordial germ cells (PGCs—right). Somatic fated cells acquire distinct methylomes according to their lineage but maintain high global levels of DNA methylation. PGCs initiate a phase of comprehensive DNA demethylation, which is complete by approximately  E12.5, and which enables subsequent establishment of a unique gamete-specific methylome during gametogenesis. Mature gametes can then fuse to form the zygote and initiate a new life cycle. ICM, inner cell mass.

DNA methylation undergoes dynamic remodelling during early embryogenesis to initially establish a globally demethylated state and then subsequently, a progressively lineage-specific methylome that maintains cellular identity and genomic stability (figure 1). The process of extensive 5mC erasure begins in the zygote following fertilization and involves both conversion to 5hmC and direct passive depletion of 5mC [39]. Reprogramming of CpG methylation culminates in a globally demethylated genome in the inner cell mass (ICM) of the pre-implantation embryo (by approx. E3.5 in mice), and correlates with the establishment of pluripotent cells, which can form embryonic stem (ES) cells *in vitro* [17,40]. Indeed, ES cells completely lacking DNA methylation are viable and competent for self-renewal, indicating DNA methylation is dispensable for the naive ground state [41]. However, DNA methylation-deficient ES cells undergo apoptosis upon *in vitro* differentiation and *Dnmt1*-null embryos die around approximately E8.5, suggesting that 5mC is a critical requirement to direct and maintain terminal cell differentiation in embryonic lineages [5]. Thus, the reduced genomic 5mC levels in the pre-implantation embryo generates an epigenetic state that is conducive for subsequent embryonic lineage-specification to proceed in parallel with de novo methylation to progressively lock in cellular identity.

Following 5mC erasure, de novo remethylation commences at implantation and is directed by DNMT3A and DNMT3B. Several mechanisms have been proposed to account for how DNA methylation patterns are established during development. These can be broadly divided into (i) interactions with chromatin modifications, (ii) protection by DNA-binding factors at autonomous DNA sequences (including CpG-density), (iii) transcription read-through, and (iv) small RNA guides [42–48]. Notably, these mechanisms probably function synergistically, at least to some extent, but also in a locus and context-dependent manner. In general, DNA methylation is the default state and variation in 5mC patterns is generated through protection of genomic regions, such as CGIs, from de novo activity. Recent studies using meDIP and RRBS have demonstrated that the bulk of de novo methylation initiates in the early ICM and is largely complete by approximately E6.5 [17,30]. Consistent with this, there is an accumulation of DNA methylation at a significant number of promoters during ES cell differentiation towards neuronal progenitors [49]. However, upon subsequent terminal differentiation, neuronal progenitors only acquire limited additional 5mC, indicating that the majority of promoter CpG methylation occurs as an early developmental step, possibly to define initial lineage-restriction rather than the terminal fate. Indeed, differential DNA methylation defines lineage-restriction to either myeloid or lymphoid progenitors during haematopoiesis [50] and DNMT3A-dependent activity is essential for haematopoietic differentiation [51]. Moreover, methylation of the *Elf5* promoter is thought to be an early epigenetic barrier between trophectoderm and embryonic lineages [52]. Thus, the progressive acquisition of developmental DNA methylation at key promoters underpins Waddington's image of canalization of cell fate, particularly, the early developmental decisions.

While the majority of studies on DNA methylation patterns during development have focused primarily on promoters, it is likely that dynamic 5mC at genic, intergenic or repeat elements also contributes to lineage restriction and the observed phenotypes in DNMT-deficient systems. Indeed, only a relatively modest number of promoters, mainly of intermediate CpG-density, exhibit lineage-dependent de novo methylation during development, implying that DNA methylation is also developmentally relevant in other genomic contexts [9,30]. The recent identification of dynamically regulated low-methylated regions (LMR) at cell-type specific enhancers supports this possibility [44], as does the observation of differential methylation at intragenic CGIs [53]. Moreover, the accumulation of DNA methylation at transposable and repeat elements likely plays a key role in maintaining genomic stability during development [54,55].

An exception to the generally unidirectional process of coupled differentiation and de novo methylation during development occurs in PGCs, where a second comprehensive reprogramming event during the life cycle erases global 5mC (discussed below). This process is essential for erasure of parent-of-origin dependent genomic imprints and underpins the acquisition of genomic plasticity [56]. Following reprogramming, PGCs enter a phase of global remethylation which results in mature gametes that exhibit either a highly methylated (sperm) or a partially methylated (oocyte) genome [17,57] (reviewed in [58]). This second phase of 5mC reprogramming results in the establishment of a unique epigenome in PGCs and enables differentiation to mature gametes, which ultimately fuse to restart the mammalian life cycle.

## 4. Reprogramming DNA methylation

**Figure 2.**
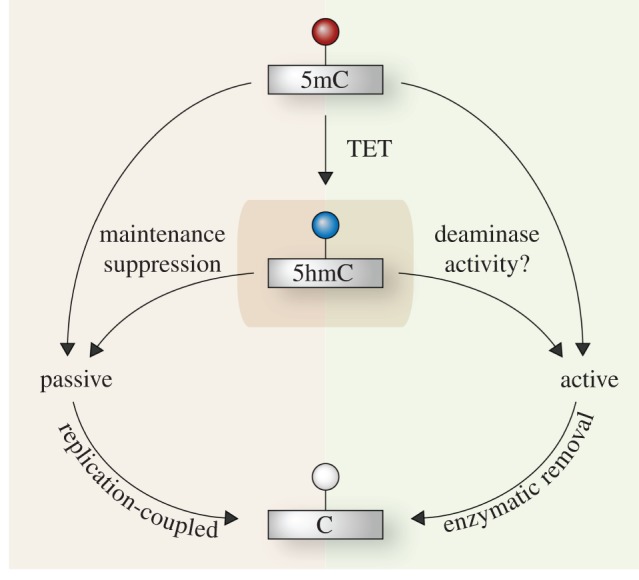
Mechanisms of DNA demethylation. Cytosine methylation (5mC) can be demethylated towards unmodified cytosine (C) through several potential mechanisms that can be broadly described as passive (left) or active (right). Passive demethylation occurs via replication-coupled dilution owing to a lack of re-establishment of DNA modification on the new daughter strand after DNA synthesis. Active erasure occurs through direct enzymatic removal of a DNA modification independent of DNA replication, and can involve a deamination reaction. Conversion to 5-hydroxymethylcytosine (5hmC) by TET proteins is likely a hub for demethylation (central box) and can lead to either passive or active erasure of DNA methylation. Additionally, 5hmC can be further modified to 5fC and 5caC (not shown), which can subsequently be actively or passively erased. 5mC can also be directly depleted passively, and potentially actively following deamination without conversion to 5hmC (upper arrows).

DNA methylation acts as a lineage-restricting barrier during development and it is therefore essential to reprogramme the stable 5mC mark to reset the life cycle for each new generation. The mechanisms that direct this process are of great interest and have recently begun to be unravelled (figure 2). In the zygote, DNA demethylation is mechanistically compartmentalized, with the maternally and paternally derived genomes undergoing distinct processes of 5mC erasure. Ultimately, this leads to a highly demethylated epigenome, with the exception of imprinted loci, rare maternally derived promoters and some transposable elements (TEs) including ETn and  intracisternal A particle (IAP) [30,59]. Original studies demonstrated that the paternal genome becomes globally demethylated prior to DNA replication, while the maternal genome apparently retains 5mC, with a subsequent progressive depletion over cell divisions [60,61]. This led to the suggestion that the paternal and maternal genomes undergo active and passive DNA demethylation, respectively. However, recent studies have demonstrated that at a global level paternal 5mC is converted to 5hmC, which is subsequently removed via passive replication-coupled dilution [39,62,63]. The paternal conversion to 5hmC is driven by the hydroxylase TET3, which can also further modify 5hmC to 5fC and 5caC [39,64]. Notably, maternal knockout of *Tet3* leads to embryonic death in a subset of embryos suggesting the conversion to 5hmC is important, if not essential, to overcome the epigenetic barriers imposed by 5mC [65]. Thus, it appears that the bulk of zygotic demethylation is induced through passive replicative-dilution, but through different mechanisms on the paternal and maternal genomes. This difference may be related to the necessity to maintain genomic imprints through reprogramming, which are primarily located on the maternal genome [66]. Paternal imprints may be maintained via an alternative mechanism based on their association with repeat elements [67]. Interestingly, both the paternal and maternal genomes exhibit a dramatic decrease in 5mC, including at imprints, in mouse zygotes deficient for STELLA. This implies that the maternal genome is protected from 5hmC conversion, rather than the paternal genome being specifically targeted in normal zygotes [68]. However, it is unclear precisely how the direct passive loss of 5mC on the maternal genome occurs, as at least some DNMT1 is present and sufficient to maintain imprints [69].

The most comprehensive DNA methylation reprogramming event in the mammalian life cycle occurs in PGCs between approximately E8.5–E12.5 [70,71]; (reviewed in [56]). This process leads to almost complete genome-wide DNA demethylation by E13.5 [72]. Importantly, 5mC erasure in PGCs includes genomic imprints, which enables their subsequent re-establishment according to the sex of the cell, and ultimately the acquisition of totipotency upon fertilization [71]. Additionally, most repeat elements are demethylated to some extent in PGCs, although IAP elements are partially resistant, which may enable transgenerational epigenetic inheritance [72–74]. The mechanistic basis of DNA demethylation in PGCs has yet to be fully determined, but could potentially occur through a number of overlapping routes including: conversion to 5hmC coupled with passive depletion, direct replication-dependent loss of 5mC, or active erasure associated with DNA repair pathways (figure 2) [56]. Given the high expression of *Tet1* and *Tet2* in PGCs, a demethylation mechanism involving 5hmC seems probable [75]. Moreover, both TET1 and TET2 may contribute to this process redundantly, as supported by only a modest reduction of 5hmC levels in *Tet1*-null ES cells (which also express *Tet2*) and the fertility, albeit sub-fertility, of *Tet1*-null mice [76]. Other mechanisms may also operate in conjunction with 5hmC conversion to drive DNA demethylation in PGCs, possibly including base excision repair (BER) systems because loss of the deaminase AID leads to a partial reduction of 5mC erasure [72,75,77]. Further studies are required to elucidate the mechanisms fully.

While the two primary phases of global 5mC erasure during the life cycle occur in pre-implantation embryos and in PGCs, significant DNA demethylation has also been reported in other contexts. A significant reduction at all genomic loci examined, except imprinted loci, has been shown using an RRBS approach during mouse erythropoiesis [78]. It is unclear how this global demethylation occurs mechanistically, but it may be linked to the requirement to activate lineage-specific genes. Indeed, despite the common view that once established, DNA methylation is stably maintained throughout development, several examples of locus-specific demethylation have emerged outwith of global reprogramming phases, particularly in neuronal and haematopoietic lineages. During terminal differentiation, the CpG-dense promoters of the eye-specific genes *Cplx4* and *Cryaa* are demethylated, possibly to enable their subsequent expression. Similarly, promoter methylation at *Tlr6* and *Cytip*, which is established by E6.5, is subsequently erased during further embryonic differentiation towards haematopoietic lineages [30]. At low CpG-density promoters, the presence of activating signals alone may be sufficient to initiate dynamic DNA demethylation [79,80]. Moreover, physiological factors such as increased muscle activity may drive dynamic 5mC erasure at key metabolic promoters in skeletal muscle [81]. Recent studies have also suggested that cyclical demethylation may contribute to the generally unmethylated state of CGIs during all stages of development. In this model TET1, which preferentially binds CpG-rich sequences, is targeted to CGIs where any aberrant 5mC could be hydroxylated and removed via subsequent passive or active mechanisms [13,26,82]. This continuous turnover could thus be considered as reiterative demethylation to maintain CGIs in a permissive state for transcription. Taken together, 5mC reprogramming appears to occur both locus-specifically and globally at least to some extent, during normal cell differentiation, although this may be specifically linked to lineage gene-activation.

## 5. The genomic role of DNA methylation

**Figure 3.**
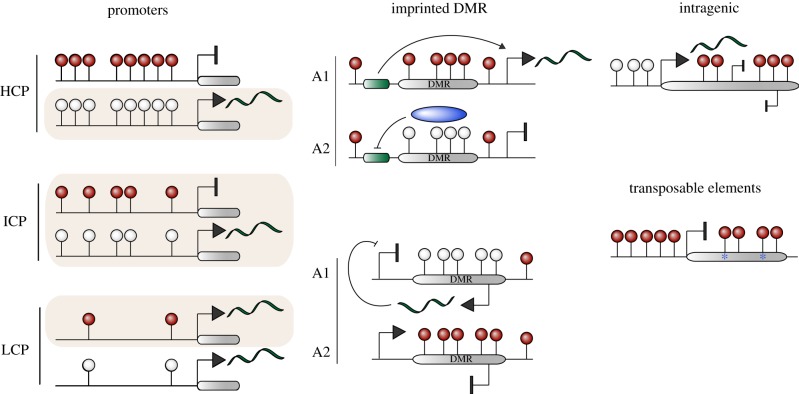
Distribution and roles of DNA methylation. The distribution of DNA methylation varies according to genomic landmarks. High-CpG density promoters (HCP) are usually hypomethylated, low CpG-density promoters (LCP) are usually methylated, and intermediate CpG-density promoters (ICP) can be either methylated or unmethylated (shown as shaded). In general, methylation only has a significant transcriptional effect at HCPs and ICPs, whereas methylation at LCPs does not correlate with repression. Note also that the absence of methylation only generates a permissive state for transcription and does not necessarily result in gene activity. Imprinted loci are methylated on one allele and hypomethylated on the other. This can have allele-specific effects by either modulating interactions between enhancers (green) and promoters (upper imprinted gene) or regulating expression of an antisense ncRNA, which silences genes in *cis* (lower imprinted gene). Gene bodies are generally hypermethylated, which may function to repress cryptic internal promoters. Transposable elements are usually highly methylated in the promoter and coding regions, which silences their expression and can lead to their mutation and inactivation through cytosine deamination (asterisks), respectively. A1/A2: Allele 1 and Allele 2. DMR, differentially methylated region.

At the cellular level DNA methylation is essential to propagate lineage restriction and to maintain genomic stability. However, at the molecular level the role of 5mC is dictated by multiple parameters, including its genomic context—such as promoter or exon, the local CpG density and local chromatin modifications [10,83]. While DNA methylation is generally considered to impede and repress transcription, genome-wide studies have demonstrated that the actual transcriptional effect of DNA methylation at promoter regions is related to the local CpG-density (figure 3) [9,84]. For example, DNA methylation at low CpG-density promoters (LCP) is not correlated with transcriptional silencing, and indeed most LCPs are methylated irrespective of their expression state. In contrast, significant 5mC at high CpG-density promoters (HCP) is strongly associated with gene silencing, although HCPs are very rarely methylated during normal development, excepting imprinted genes and the inactive X-chromosome. Thus, concerning transcriptional regulation, DNA methylation primarily functions at intermediate CpG-density promoters (ICP), which have a greater capacity to acquire lineage-dependent methylation and which are also correlated with strong gene silencing when methylated [9,30]. Of particular prominence is a role for DNA methylation in stably silencing ICPs associated with germline-specific genes [9,85,86], which may serve to stringently prevent their expression in somatic cells owing to their potential for contributing to carcinogenesis [87,88]. Further studies have shown that promoter methylation at germline-specific genes is indeed uniquely associated with their transcriptional regulation [89]. At other promoters, principally lineage- or pluripotency-specific genes, CpG methylation may function as a secondary ‘silencing lock’ that is acquired after other chromatin modifications have established a repressive state [90]. However, CpG methylation is generally not a regulatory mechanism that causally directs repression at most promoters during normal development. Indeed, it has even been reported to mediate gene activation in some rare contexts [91].

In contrast to its secondary role at most promoters, CpG methylation has a key role in modulating genomic imprinting and in transcriptional silencing of CpG-dense promoters on the inactive X-chromosome in females. Imprinted genes are expressed monoallelically according to parental origin, a process that in most cases is directly linked to differential methylation of a CpG-dense region (DMR) [92,93]. Some DMRs regulate the monoallelic expression of multiple linked genes, as demonstrated by genetic interventions, in which case the DMR is known as an imprinted control region (ICR). In general, DNA methylation at DMRs functions through two mechanisms: (i) impeding binding of a chromatin insulator protein, thereby enabling differential interactions between enhancers and promoters according to methylation state or (ii) acting as a promoter for an antisense ncRNA, which represses *cis*-linked genes when expressed from the unmethylated allele (figure 3) [94]. The deregulation of imprinted genes may significantly contribute to the phenotype of DNA methylation-deficient systems, as imprinted genes play an essential role in development, growth, postnatal behaviour and cell metabolism.

The role of intragenic DNA methylation in mammals has recently emerged as a key point of interest, since both intronic and particularly exonic sequences tend to be highly methylated. One possible role of intragenic 5mC is to prevent spurious transcriptional activation from cryptic internal promoters [10]. Alternatively, genic methylation may simply be derived from the relatively open chromatin state of active genes owing to transcription read-through, which could enable DNMTs or chromatin-modifying enzymes enhanced access. This is supported by observations that the active X-chromosome (X_a_) in females is more highly methylated in gene-bodies than the inactive X-chromosome (X_i_) [95]. Another point of interest is DNA methylation at regions adjacent to promoter CGIs, termed CpG-shores. Here, 5mC has been correlated with gene expression and correspondingly, CpG-shores are reported to be methylated tissue-specifically [50,96]. However, using an affinity-based approach, it has also been suggested that CpG-shore methylation shows no association with gene expression and indeed, that they do not exhibit significant levels of differential DNA methylation [53]. Thus, the role of CpG-shore methylation in genome regulation will require further investigation.

Finally, DNA methylation is essential for stable silencing of some TEs. In mammals retrotransposons and repeat-derived elements are usually highly methylated, and loss of 5mC can lead to significant upregulation of at least IAP and LINE elements, which can potentially lead to insertional mutagenesis [54,55]. The potential significance of 5mC in maintaining genomic integrity by silencing TEs has led to the ‘genome defence’ hypothesis that TE repression is *the* central role of DNA methylation in mammals [97], although this has been disputed [10]. Notably, the function of DNA methylation in TE silencing has been proposed to be directly coupled to its unique role in regulating a subset of germline-specific genes. Here, it has been noted that genes involved in post-transcriptional genome-defence against TEs in the germline, such as *Tex19.1* [98], are preferentially regulated by promoter CpG methylation [89]. Because these genes are activated by erasure of promoter 5mC during epigenetic reprogramming in PGCs, they become expressed at precisely the time when TEs might become transcriptionally active, owing to loss of global 5mC. Thus, by linking DNA methylation to both TE silencing and regulation of genome defence genes, a failsafe system is created whereby the potential for TE expression during 5mC loss in PGCs is coupled to upregulation of mechanisms that suppress TE activity.

## 6. Concluding remarks

As an epigenetic mechanism, DNA methylation is both elegant and enigmatic. While we understand both the mechanism that faithfully propagates CpG methylation through cell divisions and the genomic distribution of this modification, the dynamics and the mechanisms of reprogramming this mark remain to be fully elucidated. Likewise, while DNA methylation appears to carry out distinct roles in different genomic and developmental contexts, we have not precisely defined how CpG methylation contributes to genome regulation and development. Emerging technologies that can comprehensively map cell-type specific DNA methylation patterns could potentially enable a fuller understanding of 5mC in normal development, cancer, ageing and inter-individual variations. The ability to profile methylation in rare or inaccessible cell populations such as in PGCs is integral to this process. Downstream analysis of key mediators of DNA methylation patterns such as TET proteins, and integration of chromatin and transcription datasets may shed further light on the roles and dynamics of this key epigenetic modification.
